# Molecular interactions of miR-338 during tumor progression and metastasis

**DOI:** 10.1186/s11658-021-00257-w

**Published:** 2021-04-07

**Authors:** Meysam Moghbeli

**Affiliations:** Department of Medical Genetics and Molecular Medicine, School of Medicine, Mashhad University of Medical Sciences, Mashhad, Iran

**Keywords:** Non-coding RNA, MicroRNA, MiR-338, Cancer, Biomarker

## Abstract

**Background:**

Cancer, as one of the main causes of human deaths, is currently a significant global health challenge. Since the majority of cancer-related deaths are associated with late diagnosis, it is necessary to develop minimally invasive early detection markers to manage and reduce mortality rates. MicroRNAs (miRNAs), as highly conserved non-coding RNAs, target the specific mRNAs which are involved in regulation of various fundamental cellular processes such as cell proliferation, death, and signaling pathways. MiRNAs can also be regulated by long non-coding RNAs (lncRNAs) and circular RNAs (circRNAs). They are highly stable in body fluids and have tumor-specific expression profiles, which suggest their suitability as efficient non-invasive diagnostic and prognostic tumor markers. Aberrant expression of miR-338 has been widely reported in different cancers. It regulates cell proliferation, migration, angiogenesis, and apoptosis in tumor cells.

**Main body:**

In the present review, we have summarized all miR-338 interactions with other non-coding RNAs (ncRNAs) and associated signaling pathways to clarify the role of miR-338 during tumor progression.

**Conclusions:**

It was concluded that miR-338 mainly functions as a tumor suppressor in different cancers. There were also significant associations between miR-338 and other ncRNAs in tumor cells. Moreover, miR-338 has a pivotal role during tumor progression using the regulation of WNT, MAPK, and PI3K/AKT signaling pathways. This review highlights miR-338 as a pivotal ncRNA in biology of tumor cells.

## Background

Cancer is the leading cause of deaths and remains a significant challenge for human health globally. Improving the quality of life and prolonging patient survival are the main aims of cancer therapy. However, low efficiency of conventional treatments highlights the importance of effective tumor-specific therapeutic strategies [1]. Non-coding RNAs (ncRNAs) are important post-transcriptional regulators that affect cell apoptosis, proliferation, and differentiation [2–4]. MiRNAs are a group of short ncRNAs (19–24 nucleotides) that target mRNAs using binding with the 3′-untranslated region (UTR), resulting in mRNA degradation and translational suppression [5]. They are categorized based on their genomic origin into intergenic, intronic, and exonic. Aberrant expression of miRNAs has been reported during tumor progression [6, 7]. Based on the targeted mRNA, miRNAs can function as tumor suppressors or oncogenes [8, 9]. Clarification of miRNA mechanisms during pathogenesis of human disorders paves the way for introducing novel miRNA-based therapeutic modalities or diagnostic markers. MiRNAs are tissue specific and resistant to plasma RNases, which suggests their use as unique non-invasive diagnostic tumor markers [10]. Since the majority of tumors are detected in advanced stages with a poor prognosis, non-invasive and sensitive markers are required for early stage tumor detection. Circulating miRNAs are highly stable in body fluids and have tumor-specific expression profiles, suggesting their suitability as efficient noninvasive diagnostic and prognostic tumor markers [11, 12]. MiR-338 is located within an intron of the *AATK* gene that has an essential role during neuron development [13]. In the present review, we have summarized all of the reported miR-338 targets and associated signaling pathways and other non-coding RNAs to clarify the molecular mechanisms of miR-338 during tumor progression in different cancers (Table 1).

**Table 1:** molecular targets of miR-338 during tumor progressions

Study	Year	Type	Gene	Target	Samples	Function
Liu [20]	2018	Lung	Circ-0001649	miR-338-5p	53 NT^*^ A549, H358, H1299, and H1581 cell lines	Inhibited cell proliferation and migration
Zhu [24]	2020	Renal	Circ-AKT1	miR-338-3p	70 NT 768-O, A498, ACHN, Caki-1, and OS-RC-2 cell lines	Induced cell proliferation, migration, and EMT
Wang [28]	2020	Myeloma	Circ-0007841	miR-338-3p	41 patients and 41 healthy controls (plasma) H929 and OPM2 cell lines	Induced cell proliferation
Du [32]	2020	Colorectal	Circ-RAE1	miR-338-3p	80 NT HCT116, SW620, HT29, and SW480 cell lines	Induced cell migration
Xu [35]	2020	Lung	Circ-0000326	miR-338-3p	100 NT A549, SPC-A1, H1299, and H1975 cell lines	Induced tumor progression
Shu [38]	2020	Thyroid	Circ-HIPK3	miR-338-3p	10 NT K1, CAL-62, and TPC-1 cell lines	Induced progression and invasion
Liu [39]	2020	Liver	Circ-104566	miR-338-3p	87 NT SK-HEP-1, HLE, SNU449, Hep-3B, and Huh7 cell lines	Induced tumor progression
Xiong [41]	2019	Glioma	Circ-SMO742	miR-338-3p	10 NT A172 and U-87MG cell lines	Induced cell proliferation
Pu [43]	2020	Liver	Circ-0000092	miR-338-3p	40 NT Hep3B, LM3, MHCC97L, Sk-hep1, and HepG2 cell lines	Induced tumor progression
Qian [47]	2020	Cervical	Circ-HIPK3	miR-338-3p	45 NT HeLa, CaSki, SiHa, C-33A, C-4I, and SW756 cell lines	Induced EMT
Jin [48]	2020	Gastric	Circ-HIPK3	miR-338-3p	31 NT MGC803 and BGC823 cell lines	Induced cell migration
Xiang [49]	2020	Osteosarcoma	Circ-CCDC66	miR-338-3p	12 NT SW1353 and U2OS cell lines	Induced tumor invasion
Yang [50]	2020	Colorectal	Circ-0137008	miR-338-5p	30 NT HT29, HCT116, HCT8, LOVO, SW480, and SW620 cell lines	Inhibited cell proliferation and migration
Yan [56]	2017	Esophageal	SNHG1	miR-338-3p	56 NT 6 primary cell culture	Induced cell proliferation
Zhang [60]	2019	Neuroblastoma	SNHG1	miR-338-3p	33 NT SK-N-SH and SK-N-AS cell lines	Induced cell proliferation and migration
Li [65]	2019	Colorectal	SNHG15	miR-338-3p	203 NT HCT116, SW620, LOVO, SW480, and 293 T cell lines	Induced cell proliferation
Zhang [66]	2019	Prostate	SNHG15	miR-338-3p	LNCAP, DU145, and PC3 cell lines	Induced cell proliferation and EMT
Liu [69]	2018	Ovarian	LINC00460	miR-338-3p	98 NT SKOV3, A2780, OVCAR, and HO-8910	Increased cell proliferation and EMT
Hu [70]	2020	Prostate	LINC00173	miR-338-3p	124 NT DU145, PC3, and LNCAP cell lines	Induced tumor cell progression
Wan [71]	2020	Glioma	LINC00525	miR-338-3p	U87, MO59K, U118, Hs683, and LN-18 cell lines	Regulated migration and EMT
Lu [72]	2020	Gastric	LINC-00689	miR-338-3p	40 NT HGC-27, SGC-7901, MGC-803, and AGS	Induced cell growth and EMT
Feng [74]	2020	Leukemia	NEAT1	miR-338-3p	32 patients 32 healthy controls KG-1, HL-60, THP-1, and U937 cell lines	Inhibited cell growth and migration
Jing [76]	2019	Lung	CRNDE	miR-338-3p	84 NT A549, H1299, SPCA1, and H358 cell lines	Induced tumor progression
Song [79]	2020	Esophageal	BANCR	miR-338-3p	40 NT KYSE450 and KYSE510 cell lines	Inhibited tumor progression
Luan [80]	2018	Cervical	XLOC-006390	miR-338-3p	20 NT SiHa, HeLa, CaSki, C-41, and C-33A cell lines	Induced tumor progression
Chen [82]	2020	Lung	SBF2-AS1	miR-338-3p	56 NT A549, H1650, and H1975 cell lines	Induced tumor growth
Ji [83]	2019	Liver	DSCAM-AS1	miR-338-3p	48 NT HepG2, Hep3B, Huh7, and SMMC7721 cell lines	Induced tumor progression
Li [85]	2019	Oral	OIP5-AS1	miR-338-3p	38 NT SCC9, SCC15, Ca9-22, and HSU3 cell lines	Increased cell proliferation, migration, colony formation, and in vivo growth
Ma [89]	2018	Glioma	miR-338-5p	FOXD1	130 NT U251 cell line	Inhibited cell proliferation
Hua [90]	2017	Cervical	miR-338-3p	MACC1	67 NT HeLa and CaSki cell lines	Inhibited cell proliferation
Jia [91]	2019	Osteosarcoma	miR-338-3p	RUNX2, CDK4	MG-63 and U2OS cell lines	Inhibited cell proliferation and migration
Peng [96]	2014	Gastric	miR-338-3p	NRP1	41 NT SGC-7901, HGC-27, AGS, MKN-45, and N87 cell lines	Inhibited cell proliferation and migration
Ding [98]	2019	Lung	miR-338-3p	NRP1	55 NT A549, HCC827, and H226 cell lines	Inhibited colony formation and cell migration
Liu [99]	2015	Oral	miR-338-3p	NRP1	24 NT TCa-8113 and SCC-15 cell lines	Inhibited cell proliferation and invasion
Song [112]	2018	Gastric	miR-338-3p	EPHA2	AGS cell line	Inhibited proliferation and migration
Niu [116]	2019	Ovarian	miR-338-3p	WNT2B	54 NT SKOV3, A2780, and IOSE-80 cell lines	Induced cisplatin response
Xue [120]	2014	Colorectal	miR-338-3p	SMO	40 NT HT29, LOVO, Caco2, and SW-620 cell lines	Inhibited cell migration
Huang [121]	2011	Liver	miR-338-3p	SMO	36 NT Hep3B, SK-HEP-1, Huh7, Bel-7402, and SMMC-7721 cell lines	Inhibited tumor invasion
Guo [124]	2014	Gastric	miR-338-3p	PREX2a	53 NT AGS and BGC-823 cell lines	Inhibited tumor progression
Chen [125]	2013	Neuroblastoma	miR-338-3p	PREX2a	18 NT SH-SY5Y cell line	Inhibited cell proliferation and migration
Besse [128]	2016	Glioblastoma	miR-338-5p	NDFIP1, PPP2R5a	40 NT A172, T98G, and U87MG cell lines	Inhibited cell proliferation
Liu [132]	2019	Lung	miR-338-3p	AKT	A549 cell line	Inhibited cell proliferation
Sui [133]	2017	Thyroid	miR-338-3p	AKT3	48 NT 8505c, TPC-1, and SW1736 cell lines	Inhibited cell proliferation, migration, and in vivo growth
Lu [137]	2018	Cervical	miR-338-3p	ATF2	30 NT Siha, HeLa, C33A, and Me180 cell lines	Inhibited autophagy
Chu [141]	2019	Colorectal	miR-338-5p	PIK3C3	66 NT SW480 and HCT166 cell lines	Induced tumor invasion
Zhang [145]	2017	Lung	miR-338-3p	IRS2	40 NT A549, H1299, SPCA1, and H358 cell lines	Inhibited cell proliferation and migration
Wang [147]	2015	Liver	miR-338-3p	FOXP4	30 NT HepG2 and Hep3B cell lines	Inhibited cell proliferation
Tong [152]	2017	Renal	miR-338-3p	SOX4	48 NT 786-O, ACHN, Caki-1, and Caki-2 cell lines	Inhibited colony formation and cell migration
Jin [154]	2015	Breast	miR-338-3p	SOX4	32 NT MCF-7, MDA-MB-231, BT-549, and MDA-MB-453 cell lines	Inhibited colony formation, in vivo growth, and cell migration
Huang [155]	2015	Gastric	miR-338-3p	ZEB2	20 NT AGS, MKN-45, and NCI-N87 cell lines	Inhibited EMT
Lu [157]	2019	Colorectal	miR-338-3p	MACC1	15 NT SW480 and 293 T cell lines	Inhibited cell proliferation and migration
Zhang [158]	2019	Melanoma	miR-338-3p	MACC1	60 NT A375 and G361 cell lines	Inhibited cell proliferation and migration
Zou [159]	2018	Colorectal	miR-338-3p	MACC1	98 NT SW480, SW620, HT29, HCT116, and SW1116 cell lines	Inhibited tumor progression
Zhang [160]	2019	Ovarian	miR-338-3p	MACC1	105 NT	Inhibited cell proliferation and migration
Shang [161]	2016	Glioma	miR-338-3p	MACC1	39 T and 17 N U251 and U87 cell lines	Induced cisplatin sensitivity
He [166]	2020	Breast	miR-338-3p	ZEB1	148 NT MCF7 and HCC1937 cell lines	Inhibited cell proliferation and EMT
Shan [167]	2015	Nasopharyngeal	miR-338-3p	*HIF1A*	5 NT CNE-1, CNE-2, 5-8F, and 6-10B cell lines	Inhibited cell migration and proliferation
Xu [168]	2014	Liver	miR-338-3p	*HIF1A*	15 NT HepG2, SMMC-7721, BEK-7402, Hep3B, and Huh7 cell lines	Induced sorafenib response
He [170]	2020	Lung	miR-338-3p	NFATc1	20 NT A549, H1650, SPCA-1, H460, H226, and H1299 cell lines	Inhibited cell proliferation
Zhang [174]	2019	Bladder	miR-338-3p	ETS1	39 NT J82, 5637, and T24 cell lines	Inhibited cell proliferation and EMT
Li [176]	2018	Glioma	miR-338-5p	TSHZ3	35 NT U87 and U251 cell lines	Increased invasiveness
Wen [178]	2015	Ovarian	miR-338-3p	RUNX2	54 NT SKOV3, OVCAR3, and A2780 cell lines	Inhibited cell proliferation, migration, and in vivo growth
Duan [181]	2019	Breast	miR-338-3p	MORC4	30 NT MCF7 and MDA-MB-231 cell lines	Inhibited cell migration
Li [185]	2013	Gastric	miR-338-3p	SSX2IP	66 NT SGC-7901, NCI-N87, BGC-823, AGS, KATO-III, SNU-1, MKN-28, and MKN-45 cell lines	Inhibited cell proliferation and in vivo growth
Lei [191]	2017	Glioblastoma	miR-338-5p	EFEMP1	15 NT U251, A172, U-118, and U87 cell lines	Inhibited cell proliferation and migration
Chen [197]	2016	Lung	miR-338-3p	ITGB3	115 NT A549, NCI-H292, NCI-H460, NCI-H446, and NCI-H1299 cell lines	Inhibited tumor invasion
Li [200]	2018	Renal	miR-338-3p	KIFC1	58 NT 786-O, 769-P, and OS-RC-2 cell lines	Inhibited cell proliferation and migration
Cao [202]	2018	Osteosarcoma	miR-338-3p	AHSA1	20 NT MG-63, Saos2, and HOS cell lines	Inhibited EMT and invasion
Xiao [207]	2018	Liver	miR-338-3p	SPHK2	39T and 21N HepG2, SMMC-7721, and BEL7402 cell lines	Inhibited cell proliferation
Zhang [209]	2017	Lung	miR-338-3p	SPHK2	34 NT H460, H1299, A549, SPC-A-1, and Calu-3 cell lines	Inhibited cell proliferation
Han [216]	2017	Glioblastoma	miR-338-3p	PKM2	U87, LN229, and SNB19 cell lines	Inhibited cell proliferation
Zhang [217]	2016	Ovarian	miR-338-3p	PKM2	HO-890, A2780, CoC1, CoC2, SKOV3, and Caov-3 cell lines	Inhibited cell proliferation
Sun [220]	2018	Gastric	miR-338-3p	PTP1B	12 NT MKN-45 and MGC-803 cell lines	Inhibited cell proliferation

*Normal and Tumor tissues

### Circular RNAs

CircRNAs are generated by back-splicing characterized by a closed loop [14]. They are involved in transcriptional regulation by microRNA sponging, which is critical in tumor cell proliferation, apoptosis, angiogenesis, and metastasis [15, 16]. Lung cancer is the second most frequent and leading cause of cancer-related deaths in the world [17, 18]. The majority of lung tumors are non-small cell lung cancer (NSCLC) cases [19]. Circ_0001649 is a circRNA transcribed from *SHPRH*. It was reported that there was *circ_0001649* down regulation in NSCLC tissues and cell lines, which was correlated with advanced TNM stage and lymph node involvement. It inhibited cell proliferation and migration of NSCLC cells through miR-338-5p sponging [20]. Renal cell carcinoma (RCC) accounts more than 90% of renal cancers [21]. Clear cell RCC (ccRCC) is the most frequent histological type of RCC [22]. Caveolin-1 (CAV1) is a scaffold protein that interacts with G-protein and is involved in regulation of WNT and TGFβ signaling pathways [23]. It was reported that there was *circAKT1* upregulation in ccRCC tissues in comparison with normal margins, which was associated with poor prognosis, TNM stage, and lymph node involvement. *CircAKT1* induced cell proliferation, migration, and epithelial-mesenchymal transition (EMT) via miR-338-3p inhibition, which resulted in CAV1 upregulation [24]. Bromodomain containing 4 (BRD4) is a chromatin reader protein and transcriptional regulator that binds acetylated histones during cell proliferation [25–27]. It was reported that there was *circ-0007841* up-regulation in multiple myeloma (MM). It also induced cell proliferation and metastasis while suppressing apoptosis in MM cells via miR-338-3p decoy to up-regulate BRD4 [28]. *TYRO3* is a tyrosine kinase significantly upregulated in various tumors, and induces cancer cell proliferation, EMT, and drug resistance [29–31]. There was an increased level of *circRAE1* in CRC tissues that was correlated with advanced TNM stage, tumor size, and lymph node involvement. It also promoted colorectal tumor cell migration by miR-388-3p sponging to upregulate *TYRO3* [32]. *RAB14* is an oncogenic GTPase involved in cell proliferation and invasion through regulation of the AKT pathway [33, 34]. There was significant *circ-0000326* upregulation in lung cancer tissues compared with normal margins. It regulated lung tumor cell proliferation and migration via *RAB14* upregulation following miR338-3p sponging. There were also associations between *circ-0000326* upregulation, T stage, N stage, and tumor grade of differentiation [35]. *RAB23* belongs to the Rab GTPase family involved in vesicular transportation and endocytosis. It functions as an oncogene in many solid tumors such as bladder (BCa) and prostate (PCa) cancers. RAB23 induced BCa cell proliferation via the NF-κB and integrin β1/Rac1 axis, and induced cisplatin resistance by the Shh-Gli-ABCG2 axis [36, 37]. It was reported that *circHIPK3* silencing significantly reduced thyroid cancer (TC) cell proliferation and migration. It upregulated *RAB23* via miR-338-3p sponging in TC cells [38]. *FOXP1* is a developmental transcription factor involved in embryogenesis and tumorigenesis. There was *circ_104566* upregulation in high-grade compared with low-grade hepatocellular carcinoma (HCC) tumors, introducing a direct association between *circ_104566* and TNM stage. *Circ_104566* upregulation was also correlated with shorter overall survival that referred to the prognostic role of this marker in HCC. Circ_104566 silencing reduced HCC tumor cell progression, whereas it increased apoptosis through the miR-338-3p/FOXP1 axis [39]. Smoothened (SMO) is a G protein coupled receptor of the hedgehog signaling pathway that activates GLI1 to promote cell cycle progression and migration [40]. It was shown that there was *circSMO742* upregulation in glioma cells in comparison with normal cells, which induced cell proliferation and inhibited apoptosis via miR-338-3p sponging and *SMO* upregulation [41]. *HN1* is a negative regulator of AKT-mediated GSK3B signaling. It promotes CTNNB1 phosphorylation and degradation via GSK3B activation, resulting in APC/CTNNB1/GSK3B complex inhibition. It also suppresses the AR-signaling pathway by receptor degradation [42]. *Circ_0000092* silencing reduced the levels of *HN1* expression by miR-338-3p upregulation, which resulted in reduced HCC tumor progression. There was also *circ_0000092* upregulation while there was miR-338-3p downregulation in HCC samples [43]. Hypoxia upregulates HIFs by stabilization of *HIF1A* subunits, which has critical functions during tumor angiogenesis and chemo-radio resistance [44, 45]. *HIF1A* and hypoxia-related miRNAs are involved in clinical outcomes of cancer patients [46]. There was *circ-HIPK3* upregulation in cervical cancer (CC) cells and tissues. *Circ-HIPK3* upregulated *HIF1A* by miR-338-3p sponging in CC [47]. It was observed that *circ-HIPK3* upregulation by *HIF-2α* increased GC cell migration and invasion through the miR-338-3p/miR-653-5p/NPR1 axis. There was also *circ-HIPK3* upregulation in gastric cancer (GC) tissues in comparison with normal margins [48]. Tyrosine phosphatase functions as a regulator of protein folding in endoplasmic reticulum. It was observed that there was *circCCDC66* upregulation in osteosarcoma (OS) tissues compared with normal tissues, which was associated with relapse and metastasis. *CircCCDC66* silencing also suppressed OS cell proliferation and metastasis via the miR-338-3p/PTP1B axis [49]. CDH1 is a Ca^2+^-dependent cell adhesion factor participating in cell motility and proliferation. Significant *circ-0137008* downregulation was observed in colorectal cancer (CRC) tissues and cell lines. It sponged the miR-338-5p to inhibit colorectal tumor cell proliferation and migration through *CDH1* upregulation [50].

### Long non-coding RNA

LncRNAs are a type of ncRNAs longer than 200 nucleotides that have pivotal regulatory roles in cell proliferation, apoptosis, and angiogenesis [51, 52]. LncRNAs mainly function as competing endogenous RNAs (ceRNAs) to regulate mRNA through miRNAs sponging [53]. Cystatins are considered as inhibitors of C1 cysteine proteases in various cellular processes and disorders [54]. Cystatin C (CysC) triggers caspase-mediated apoptosis by cathepsin targeting [55]. It was reported that there was miR-338 downregulation in 30% of a sample of esophageal squamous cell carcinoma (ESCC) tissues in comparison with normal margins. Significant CysC protein upregulation was also observed in ESCC tissues that induced tumor cell growth, while inhibiting apoptosis. Moreover, there was significant *SNHG1* upregulation in ESCC tissues compared with normal margins that inhibited cell apoptosis and induced proliferation through miR-338-3p sponging [56]. Polo-like kinases (PLKs) are important regulators of various cellular processes such as the cell cycle, centrosome duplication, and cell motility [57, 58]. Polo-like kinase 4 (PLK4) is involved in centriole duplication and cell cycle progression [59]. *SNHG1* induced neuroblastoma cell proliferation and migration by miR-338-3p sponging resulting in *PLK4* upregulation [60]. *SNHG16* upregulation is also associated with drug resistance and tumor progression in various cancers [61–63]. It was reported that there was significant *SNHG16* upregulation in cisplatin-resistant NB cells and tissues. *SNHG16* upregulated *PLK4* through miR-338-3p sponging in cisplatin-resistant NB cells. The SNHG16/PLK4/miR-338-3p axis also activated the PI3K/AKT signaling pathway in NB cells [64]. *RAB14* is a member of the GTPases that has a pivotal role in intracellular membrane trafficking between Golgi and endosomes. *FOS* is a leucine zipper DNA binding protein that forms a heterodimer transcriptional complex with the *JUN* family to regulate cell proliferation, apoptosis, and differentiation. *SNHG15* upregulation was observed in CRC tissues compared with normal margins that were associated with poor prognosis. It promoted CRC cell proliferation while suppressing apoptosis by miR‐338‐3p sponging, which resulted in *FOS* and *RAB14* upregulation [65]. As a critical regulator of TGFβ signaling, *FKBP1A* prevents TGFβ receptor activation in ligand absence. There were increased levels of *SNHG15* expression in PCa cells. *SNHG15* silencing reduced PCa cell proliferation and the EMT process. It seems that *SNHG15* has oncogenic roles by regulation of the FKBP1A/miR338-3p axis in PCa cells [66].

Epithelial ovarian cancer (EOC) represents about 90% of all ovarian cancers (OC) [67]. Conventional therapeutic methods such as surgery and chemotherapy have been improved during recent years; however, there is still a poor prognosis in OC patients because of tumor metastasis and chemoresistance [68]. It was observed that there was *LINC00460* upregulation in EOC tissues and cell lines that was correlated with FIGO stage, lower survival, and lymph node involvement. *LINC00460* increased EOC cell proliferation and the EMT process partially through miR-338-3p regulation [69]. As a member of RAS GTPases, *RAB25* is involved in intracellular membrane traffic and cell survival. It has a dual function and can play an oncogenic or tumor suppressor role based on cell context. *LINC00173* increased PCa progression through *RAB25* upregulation following miR-338-3p sponging [70]. It was reported that there was *LINC00525* upregulation in glioma cell lines that regulated the tumor growth, migration, and EMT process through miR-338-3p sponging [71]. Homeobox proteins are developmental transcription factors with pivotal roles during morphogenesis and differentiation. *LINC00689* upregulation was observed in GC tissues and cell lines. *LINC00689* promoted GC cell growth and the EMT process by miR-338-3p targeting that resulted in HOXA3 overexpression [72].

*CREB3* is a leucine zipper DNA binding protein that regulates cell proliferation by binding with cAMP-response element. *CREB3* functions as a tumor suppressor of glioblastoma via inhibition of hypoxia-induced autophagy [73]. *NEAT1* upregulated *CREBRF* via miR-338-3p sponging to suppress AML cell growth and migration, while induction of apoptosis [74]. *CRNDE* is an oncogenic lncRNA involved in tumor cell proliferation and migration [75]. There was significant *CRNDE* upregulation in NSCLC tissues and cell lines, which was directly correlated with lymph node involvement, advanced stage, and shorter overall survival. *CRNDE* silencing also inhibited tumor growth in nude mice. It induced NSCLC progression via miR-338-3p sponging [76]. IGF1R/IGF interaction activates Raf/MEK/ERK and P13K/AKT/mTOR signaling pathways, which are critical processes during tumor progression [77, 78]. There was *BANCR* upregulation in ESCC cells and tissues. *BANCR* silencing reduced in vivo tumor growth, migration, and EMT via miR-338-3p sponging, which resulted in inhibition of the IGF1R/Raf/MEK/ERK pathway in ESCC cells [79]. It was reported that *XLOC_006390* inhibition significantly upregulated miR-338-3p, which resulted in *PKM2* and *EYA2* targeting in cervical tumors [80]. ADAM metallopeptidase domain 17 (ADAM17) is a metalloprotease that has pivotal roles in activation of TGFβ, MAPK, and NOTCH signaling pathways [81]. There was significant *SBF2-AS1* upregulation in NSCLC tissues and cell lines, which was correlated with distant metastasis, poor prognosis, and advanced stage. *SBF2-AS1* increased NSCLC tumor growth probably through *ADAM17* upregulation following miR-338-3p sponging [82]. There was *DSCAM-AS1* upregulation in HCC cell lines and tissues, which was correlated with poor prognosis. *DSCAM-AS1* silencing reduced HCC cell proliferation, migration, and in vivo tumor growth. *DSCAM-AS1* upregulated *CCND1* and *SOD* via miR-338-3p sponging [83]. *NRP1* is a co-receptor of VEGF functioning as an oncogene in different tumors [84]. It was reported that there was *OIP5-AS1* upregulation in oral squamous cell carcinoma (OSCC) tissue and cell lines in comparison with normal margins and cells. *OIP5-AS1* significantly increased OSCC cell proliferation, migration, colony formation, and in vivo growth by regulation of the miR-338-3p/NRP1 axis [85].

### MAPK and EGFR signaling pathways

Mitogen-activated protein kinase (MAPK) is a critical signaling pathway involved in regulation of cell proliferation, migration, differentiation, and apoptosis in response to extracellular signals [86]. Glioma tumors originate from glial progenitor or glial cells in the spine or brain [87]. *FOXD1* is considered as a developmental transcription factor [88]. It was found that miR-338-5p suppressed MAPK signaling by *FOXD1* downregulation to inhibit glioma cell proliferation and induce apoptosis [89]. MET transcriptional regulator (MACC1) is a transcription factor involved in tumor metastasis through the HGF/c-MET/MAPK axis. There was significant miR-338-3p downregulation in cervical cancer tissues that was associated with lymph node involvement, FIGO stage, and depth of invasion. MiR-338-3p inhibited MAPK signaling by *p38* and *ERK1/2* downregulation in cervical tumor cells. Generally, miR-338-3p reduced cervical tumor cell proliferation through *MACC1* targeting [90]. There were lower levels of miR‐338‐3p expressions in OS cell lines in comparison with normal cells. MiR‐338‐3p also decreased OS cell proliferation and migration, while it induced apoptosis via *RUNX2* and *CDK4* targeting and MAPK signaling pathway regulation [91]. EGFR belongs to the tyrosine kinase receptors, which are activated EGF ligands. Deregulation of EGFR signaling has been reported in tumor progression in different cancers [92]. MiRNAs are associated with drug resistance to anti-EGFR agents [93]. Neuropilin 1 (NRP1) as a coreceptor of growth factors is involved in tumor progression that affects tumor cell viability through EGFR and ErbB2 signaling pathways [94, 95]. NRP1 is widely upregulated in different solid tumors such as GC [96] and esophageal cancer [97]. It was observed that EGFR-TKI-mediated chemosensitivity was associated with the regulatory role of miR-338-3p on NRP1 in lung cancer. MiR-338-3p suppressed NSCLC colony formation and migration via *NRP1* targeting [98]. It was reported that there was miR-338-3p downregulation in oral squamous cell carcinoma (OSCC) tissues. MiR-338-3p suppressed OSCC cell proliferation and invasion via *NRP1* targeting [99]. NRP1 induces angiogenesis by p38 MAPK and ERK signaling pathways [100]. MiR-338-3p suppresses GC cell migration and proliferation, while it induces apoptosis through *NRP1* targeting [96].

### WNT and hedgehog signaling pathways

WNT signaling is a pivotal pathway that regulates various cellular processes during embryogenesis and tumorigenesis. Regarding the post-transcriptional regulatory roles of miRNAs, they can be suggested as modulators of protein components in the WNT pathway [101–103]. Gastric cancer (GC) is the most frequent gastrointestinal malignancy and the leading cause of cancer-related mortality in developing countries [104]. Despite recent progress in surgical and chemotherapeutic methods, there is still a low rate of 5-year survival due to advanced stage diagnosis [105]. *SOX5* is a developmental transcription factor [106]. Significant miR-338-3p downregulation was observed in GC tissues and cell lines, which was associated with lymph node metastasis and advanced TNM stage. There was also cross-talk between WNT and hypoxia signaling, in which hypoxia promoted WNT signaling by the HIF-a/miR-338-3p/SOX5 axis. MiR-338-3p was downregulated by *HIF1A* [107]. EMT is critical process during tumor progression and metastasis in which epithelial cells gain mesenchymal features to have better abilities for invasion. This process is characterized by E-cadherin downregulation and vimentin upregulation [108–110]. The WNT signaling pathway regulates cell proliferation, polarity and death during embryogenesis [111]. It affects cell adhesion by E-cadherin downregulation that induces cell migration and EMT. It was reported that miR-338-3p inhibited GC proliferation and migration via *EPHA2* targeting [112]. *MACC1* regulates Met expression, which is a pivotal factor of HGF/Met and master regulator of EMT [113]. HGF/MET signaling promotes migration and angiogenesis in ovarian cancer (OC) [114]. It was reported that miR-338-3p reduced OC cell proliferation and migration by *MACC1* or *MET* regulation. MiR-338-3p also downregulated *MMP2/9*, *VIM*, *CTNNB1*, and N-cadherin, while it upregulated E-cadherin, which resulted in inhibition of WNT and MEK/ERK signaling pathways [115]. WNT2B is a ligand for the frizzled family receptors that triggers the canonical WNT signaling pathway as an important regulatory pathway involved in cell proliferation and EMT. There was a correlation between cisplatin resistance and levels of miR-338-3p expression in OC patients. MiR-338-3p increased cisplatin sensitivity through *WNT2B* targeting in ovarian tumor cells [116].

The hedgehog (Hh) signaling pathway is a conserved evolutionary embryonic signal transduction pathway from extracellular signals by specific receptors such as SMO and PTCH to the nucleus where it activates Gli transcription factors. Aberrant Hh signaling is associated with tumor progression [117]. Therefore, miRNAs have critical functions in regulation of Hh signaling during tumorigenesis. Activated SMO triggers a cytoplasmic cascade to activate GLI transcription factor that regulates cell proliferation, adhesion, and differentiation [118, 119]. MiR-338-3p downregulation has been reported in CRC tissues, which was correlated with TNM stage and invasion. Advanced stage tumors had lower levels of miR-338-3p expression compared with primary stage tumors. MiR-338-3p inhibited CRC invasion and migration via SMO targeting [120]. It was reported that there was miR-338-3p downregulation in liver tumor cells. MiR-338-3p reduced liver tumor cell migration via *SMO* targeting. *SMO* and *MMP9* levels of expressions were also correlated with advanced stages in HCC tissues. Therefore, miR-338-3p inhibited HCC invasiveness by downregulation of SMO-mediated *MMP9* expression [121].

### PI3K/AKT signaling

Phosphatidylinositol 3-kinase (PI3K) is a pivotal coordinator between extracellular signals and intracellular signaling which is activated by tyrosine kinase or G-protein coupled receptors. AKT is the main effector of PI3K that is negatively regulated by PTEN [122]. PREX2a belongs to the PIP3-dependent Rac exchanger (PREX) family of proteins associated with aggressive tumors [123]. It can directly suppress the lipid phosphatase activity of PTEN, resulting in PIP3 accumulation and increased AKT phosphorylation that induces cell cycle progression and growth during tumorigenesis. There was *PREX2a* upregulation in GC in comparison with normal margins. MiR-338-3p targeted *PREX2a*, resulting in PTEN activation and reduced AKT phosphorylation. MiR-338-3p also regulated *BCL-2* and *BAX* through induction of the PI3K/AKT signaling pathway [124]. MiR-338-3p reduced neuroblastoma tumor cell proliferation and migration via *PREX2a* targeting and PTEN/AKT regulation [125]. NDFIP1 is a ubiquitin E3 ligase that regulates PTEN stability and nuclear translocation along with *RAB5*. *PTEN* has a regulatory effect on the PI3K/Akt pathway, and also on *E2F1* and *RAD51* transcriptional functions. Moreover, *PTEN* is involved in double-strand break DNA repair [126, 127]. Significant miR-338-5p and 3p downregulation was reported in glioblastoma multiforme (GBM) patients in comparison with normal controls. MiR-338-5p significantly reduced GBM cell proliferation, while promoting apoptosis through *NDFIP1* and *PPP2R5a* targeting [128]. AKT is a serine-threonine kinase that promotes tumor cell proliferation, inhibits apoptosis, and improves hypoxia resistance [129, 130]. It is involved in CREB1 phosphorylation, which has a critical role in *BCL2* and *MCL1* upregulation [131]. It was found that miR-338-3p reduced lung tumor cell proliferation by inhibition of the AKT/β-catenin pathway [132]. There was significant miR-338-3p downregulation in TC tissues compared with normal margins, which was inversely associated with stage and lymph node involvement. MiR-338-3p also significantly inhibited TC cell proliferation, motility, and in vivo growth via *AKT3* targeting .[133] It was reported that there was miR-338-5p upregulation in melanoma tissues that was conversely associated with *CD82* expression levels. MiR-338-5p induced melanoma cells proliferation and metastasis via *CD82* targeting and p-AKT upregulation [134]. Autophagy is promoted by many factors, including growth factors and DNA damage, and it regulates many pathways [135]. As a double-edged sword autophagy can suppress tumor progression in the early stages. Increased autophagy stress of tumor cells is observed following anticancer therapy, which suggests that autophagy is a cellular protection mechanism [136]. MiR-338-3p suppresses autophagy in cervical tumor cells by *ATF2* regulation via the PI3K/AKT/mTOR signaling pathway [137]. *PIK3C3* induces autophagy nucleation and inhibits EMT by degrading SNAIL and TWIST transcription factors in BC cells, leading to decreased cell migration and metastasis [138–140]. It was reported that miR-338-5p promoted CRC invasion by *PIK3C3* targeting. It also repressed autophagy and inhibited degradation of snail and twist to induce tumor progression via EMT. Moreover, there was a direct association between levels of miR-338-5p expressions and clinicopathological tumor features including advanced stages and poor survival among CRC patients [141]. IRS2 is an adaptor protein for surface receptors such as IGF-1R that activates PI3K/AKT signaling to promote cell proliferation [142–144]. It was reported that there was miR-338-3p downregulation in NSCLC tissues and cell lines in comparison with normal margins and cells, which was inversely correlated with advanced stage tumors with lymph node involvement. MiR-338-3p also reduced NSCLC cell proliferation and migration, while it increased apoptosis via *IRS2* targeting [145].

### Transcription factors and regulators

The forkhead box proteins are a family of transcription factors which are involved in regulation of cell cycle progression, cell migration, and angiogenesis. There is a complex association between miRNAs and function of forkhead box transcription factors [146]. *FOXP4* is a member of the forkhead box (FOX) transcription factors with critical roles during embryogenesis and tumor progression. There was significant miR-338-3p downregulation in HCC tissues compared with normal margins. It also inhibited HCC cell proliferation via *FOXP4* targeting [147]. *SOX4* is a developmental transcription factor and critical regulator of EMT that is overexpressed in a variety of tumors [148, 149]. It regulates the miRNA machinery and various signaling pathways such as NOTCH, TGF-β, WNT, and hedgehog [150, 151]. There was miR-338-3p downregulation in renal cell carcinoma (RCC) tissues that was correlated with lymph node involvement and TNM stage. MiR-338-3p also suppressed colony formation and migration of RCC through *SOX4* targeting [152]. EMT can be promoted by *SOX4* through *EZH2* as a polycomb epigenetic regulator [153]. MiR-338-3p was downregulated in BC tissues, which was were correlated with advanced TNM stage and lymph node involvement. MiR-338-3p suppressed breast tumor cell colony formation, in vivo growth, and migration, while promoting apoptosis via *SOX4* targeting [154].

*MACC1* is an important transcription factor involved in EMT that promotes tumor cell migration and distant metastasis [155]. It also regulates the basolateral polarity of epithelial cells and angiogenesis [156]. There was significant downregulation of miR-338-3p in CRC tissues. MiR-338-3p functions as a tumor suppressor through *MACC1* inhibition in CRC [157]. It was observed that there was miR-338-3p downregulation in malignant melanoma (MM) tissues that was correlated with advanced TNM stages and lymph node involvement. MiR-338-3p reduced MM cell proliferation and migration through *MACC1* targeting, resulting in E-cadherin upregulation and vimentin downregulation [158]. MiR-338-3p was downregulated in CRC cell lines and tissues compared with normal cells and margins, which was correlated with differentiation, TNM stage, and overall survival. It inhibited CRC progression by *MACC1* targeting [159]. Another study reported that there were significant miR-338-3p downregulation and *MACC1* upregulation in EOC tissues compared with normal tissues, which were associated with advanced stage, high grade, recurrence, and metastatic lymph nodes. EOC patients with low levels of miR-338-3p expression also had significantly shorter overall survival [160]. miR-338-3p downregulation was observed in glioma tissues, which was inversely associated with histological grades. MiR-338-3p also inhibited cell proliferation, while it induced apoptosis and cisplatin sensitivity by *MACC1* targeting [161].

Tumor hypoxia is one of the main reasons for chemoradiotherapeutic resistance, which affects tumor invasiveness and poor prognosis [162]. *HIF1A* is a transcription factor with critical functions in the hypoxia response by induction of metastasis and angiogenesis [163–165]. It was reported that there was miR-338-3p downregulation in BC tissue and cells. MiR-338-3p suppressed breast tumor cell proliferation and EMT through *ZEB2* targeting. *HIF1A* also reduced the levels of miR-338-3p expression [166]. It was found that miR-338-3p inhibited cell migration and proliferation by *HIF1A* targeting in nasopharyngeal carcinoma (NPC) cells. There was also miR-338-3p downregulation in NPC tissues [167]. Another study showed that there was significant miR-338-3p downregulation in HCC tissues compared with normal tissues. MiR-338-3p inhibited HCC cell viability, while it increased the sorafenib response and apoptosis rate through *HIF1A* targeting [168].

*NFATc1* is a DNA binding transcription complex with critical roles in stimulation of production of cytokines such as IL-2 and IL-4 in T-cells. It can also regulate activation and apoptosis of T-cells, lymphoid, and non-lymphoid cells [169]. It was reported that there was significant *NFATc1* upregulation in NSCLC tissues. *NFATc1* silencing increased the levels of *CCND1* and *CDK4* expression, whereas it downregulated *p27*. Moreover, *NFATc1* silencing upregulated *CDH1* and downregulated *CDH2* and vimentin. MiR-338 reduced cell proliferation and EMT via *NFATc1* targeting in NSCLC cells [170]. *ZEB1* is a zinc finger E-box-binding transcription factor that functions as a transcriptional suppressor in various genes such as *IL-2* and *CDH1*. It recruits the SMARCA4/BRG1 complex to represses *CDH1* during EMT [171, 172]. There was miR-338-3p downregulation in advanced GC tumors. MiR-338-3p reduced GC cell migration and EMT through *ZEB2* targeting and MACC1/MET/AKT axis inhibition. MiR-338-3p was inversely associated with *CDH2*, *MACC1*, and *ZEB2*, while directly associated with *CDH1* expression in GC samples [155]. *ETS1* is a transcription factor involved in tumor metastasis [173]. There was miR-338-3p downregulation in bladder cancer (BCa), which was correlated with TNM stage and lymph node involvement. MiR-338-3p reduced BCa cell proliferation and EMT via *ETS1* inhibition [174]. *TSHZ3* is a critical developmental transcription factor during muscle cell differentiation [175]. An inverse association was found between levels of miR-338-5p and *TSHZ3* expression in high-grade astrocytic gliomas. MiR-338-5p increased glioma invasiveness by *TSHZ3* targeting [176]. *RUNX2* is a developmental transcription factor involved in normal bone development [177]. There was significant miR-338-3p downregulation in EOC tissues compared with normal margins that was inversely correlated with lymph node metastasis, advanced FIGO stage, and grade. MiR-338-3p significantly reduced EOC cell proliferation, in vivo tumor growth, and migration through *RUNX2* targeting and inhibition of the PI3K/AKT pathway [178]. Baicalin is an anti-proliferative and anti-inflammatory alkaloid used in treatment of cancers or disorders [179]. *MORC4* functions in generation of nuclear bodies and is involved in cell cycle progression. It is also involved in chemoresistance by activation of the transcription factor *STAT3* [180]. Baicalin reduced the levels of *MORC4* following miR-338-3p upregulation, which resulted in increased apoptosis and reduced migration in BC cells [181]. Methylation is an important epigenetic mechanism during tumor progression that promotes tumor growth via hypermethylation and hypomethylation of the CpG island promoter sequences in tumor suppressors and oncogenes, respectively [182]. *MECP2* is a key member of the DNA methylation machinery involved in neural development [183]. There was also *MECP2* upregulation in GC which promoted cell proliferation in vitro/vivo. *MECP2* reduced the levels of miR-338 expression by binding with methylated CpG islands in the promoter sequence. Significant miR-338-5p downregulation was observed in GC tissues that inhibited cell growth through *BMI1* targeting as a proto-oncogene involved in transcriptional regulation [184].

### Structural and adhesion factors

*SSX2IP* is a member of the adhesion system that is involved in cell–cell adherens junctions through alpha-actinin. It also regulates cell motility in response to PDGF by activation of Rac signaling. It was found that there was miR-338-3p downregulation in GC tissues in comparison with normal margins. MiR-338-3p reduced GC cell proliferation and in vivo growth, while it induced apoptosis through *SSX2IP* targeting. MiR-338-3p downregulation was correlated with metastatic lymph nodes, larger tumor size, and advanced tumor stage [185]. EGF containing fibulin extracellular matrix protein 1 (EFEMP1) belongs to the fibulin family of extracellular matrix (ECM) glycoproteins that regulate cell adhesion, growth, and movement [186]. It also maintains tissue hemostasis by regulation of the tissue inhibitors of matrix metalloproteinase (TIMMPs) and MMPs [187, 188]. *EFEMP1* exerts its tumor suppressor activity by suppression of tumor angiogenesis and inhibition of the EGFR/AKT signaling pathway [189, 190]. It was reported that miR-338-5p was downregulated in GBM tissues. MiR-338-5p also reduced GBM cell proliferation and migration, while it significantly induced apoptosis through *EFEMP1* inhibition [191]. *ITGB3* is a receptor of various ECM proteins including fibronectin, laminin, *MMP-2*, and thrombospondin [192]. It regulates several signaling pathways, such as NRG1-ERBB, FGF1, and IGF1 signaling [193–195]. Therefore, it can be introduced as a pivotal regulator of angiogenesis, adhesion, and metastasis [196]. There was an inverse association between levels of miR-338 and ITGB3 expression in lung tumor tissues [197]. *KIFC1* is a kinesin motor protein involved in clustering of extra centrosomes in tumor cells [198]. It is required for tumor cell survival and invasion by regulation of excess centrosomes to generate progeny cells and maintenance toward apoptosis-mediated aneuploidy [199]. *KIFC1* silencing reduces RCC cell proliferation, migration, and in vivo growth by inhibition of PI3K/AKT signaling and downregulation of *MMP-2* and *VEGF*. MiR-338-3p reduced cell migration and proliferation by *KIFC1* targeting. Also *KIFC1* upregulation was significantly correlated with advanced pT and pTNM stage and larger tumor size in RCC tissues [200]. *AHSA1* is a chaperone involved in maturation, degradation, and stabilization of oncogenes [201]. There was miR-338-3p downregulation in osteosarcoma (OS) cell lines and tissues. MiR-338-3p suppressed viability, EMT, and invasion of OS cells through *AHSA1* targeting [202]. *RAB14* belongs to the RAS family of GTPases involved in intracellular membrane traffic during embryogenesis. MiR-338-3p downregulation was observed in NSCLC tissues compared with normal margins, which was directly correlated with differentiation, and conversely associated with stage and lymph node involvement. MiR-338-3p inhibited NSCLC cell proliferation, while it promoted apoptosis through *RAB14* targeting [203].

### Kinases and phosphatases

Sphingolipids are a group of hydrophobic molecules including sphingoid-base phosphates and sphingoid bases that are associated with cell proliferation and apoptosis [204, 205]. Sphingosine kinase 2 (SphK2) is involved in formation of sphingosine-1-phosphate (SPP) as a lipid mediator in cellular functions. It also participates in transcriptional regulation by *HDAC1* and *HDAC2* suppression and histone acetylation [206]. It regulates ATP and ROS levels in dopaminergic neurons. Significant miR-338-3p downregulation was observed in liver tumor tissues in comparison with normal margins. MiR-338-3p exerts its tumor suppressor role by *SPHK2* targeting in liver tumor cells [207]. MiR-338-3p downregulation was reported in colorectal and gastric cancers [155, 203, 208]. There was miR-338-3p downregulation in NSCLC tissues that was significantly associated with stage. MiR-338-3p also reduced NSCLC cell proliferation and promoted apoptosis via *SPHK2* targeting [209]. Pyruvate kinase M2 (PKM2) is responsible for ATP production via regulation of aerobic glycolysis [210, 211]. Despite the metabolic functions, *PKM2* inhibits mTOR-mediated tumor progression, and also it is involved in cell cycle regulation and drug resistance [212, 213]. *PKM2* regulates the WNT pathway by regulation of *CTNNB1* transcriptional activity and also functions as a transcriptional regulator by phosphorylation of histone H3 [214, 215]. It was reported that there was a rising trend of *PKM2* expression levels with glioma grade in which *PKM2*-overexpressed glioblastoma tumors had worse outcomes. *PKM2* ectopic expression promoted glioma cell proliferation and metabolism. MiR-338-3p suppressed glioma cell proliferation by *PKM2* targeting [216]. There was miR338-3p downregulation in OC tissues in comparison with normal margins. MiR-338-3p also suppressed cell proliferation and ATP production in OC cells through *PKM2* targeting [217]. Protein-tyrosine phosphatase 1B (PTP1B) is a tyrosine phosphatase involved in regulation of leptin and insulin signaling pathways [218]. It also functions as a tumor suppressor by dephosphorylation and inactivation of oncogenic kinases. MiR-338-3p is a brain-specific miRNA participating in regulation of axonal respiration [156, 219]. There was an inverse association between levels of miR-338-3p and *PTP1B* expression in GC tissues. MiR-338-3p exerted its tumor suppressor role through *PTP1B* targeting in GC. *PTP1B* upregulation also significantly increased the levels of pAKT and p-ERK1/2 expression in GC tumor cells [220].

## Conclusions

Since the mammalian miRNAs and their target mRNA sequences are not perfectly complementary, various mRNAs can be targeted by a specific miRNA. Therefore, there is a great challenge to clarify the precise molecular mechanisms of specific miRNAs. Understanding the molecular mechanism of miRNAs is important to introduce novel therapeutic strategies for human disorders and malignancies. However, there are contradictory reports about the role of miRNAs in different malignancies, which may be related to the use of computational and predictive methods instead of experimental verification. Moreover, the protein levels do not always exactly reflect the levels of mRNA expression. Therefore, miRNA studies should always be accompanied by protein assessments [221]. In the present review, we have summarized miR-338 target genes and associated signaling pathways to clarify the molecular mechanisms of miR-338 during tumor progression. It was observed that miR-338 mainly functions as a tumor suppressor in different cancers. There were also significant associations between miR-338 and other non-coding RNAs including circular and long non-coding RNAs in tumor cells.

## Data Availability

The datasets used and/or analyzed during the current study are available from the corresponding author on reasonable request.

## Abbreviations

miRNAs: MicroRNAs
ncRNAs: Non-coding RNAs
lncRNAs: Long non-coding RNAs
miRNAs: MicroRNAs
circRNAs: Circular RNAs
RISC: RNA induced silencing complex
EMT: Epithelial mesenchymal transition
NSCLC: Non-small cell lung cancer
RCC: Renal cell carcinoma
ccRCC: Clear cell RCC
CAV1: Caveolin-1
BRD4: Bromodomain containing 4
MM: Multiple myeloma
TC: Thyroid cancer
HCC: Hepatocellular carcinoma
SMO: Smoothened
CC: Cervical cancer
CRC: Colorectal cancer
PLKs: Polo-like kinases
PLK4: Polo like kinase 4
PCa: Prostate cancer
EOC: Epithelial ovarian cancer
ADAM17: ADAM metallopeptidase domain 17
VEGF: Vascular endothelial growth factor
EGFR: Epidermal growth factor receptor
NRP1: Neuropilin 1
GC: Gastric cancer
OC: Ovarian cancer
Hh: Hedgehog
PI3K: Phosphatidylinositol 3-kinase
PREX: PIP3-dependent Rac exchanger
FOX: Forkhead box
MM: Malignant melanoma
NPC: Nasopharyngeal carcinoma
ECM: Extracellular matrix
TIMMPs: Tissue inhibitors of matrix metalloproteinase
OS: Osteosarcoma
SphK2: Sphingosine kinase 2
SPP: Sphingosine-1-phosphate
PKM2: Pyruvate kinase M2
PTP1B: Protein-tyrosine phosphatase 1B
CysC: Cystatin C
CysC: Oral squamous cell carcinoma
CysC: Osteosarcoma
GC: Gastric cancer
MACC1: MET transcriptional regulator
EFEMP1: EGF containing fibulin extracellular matrix protein 1
